# Maternal confidence after birth in at-risk and not-at-risk mothers: internal and external validity of the Danish version of the Karitane Parenting Confidence Scale (KPCS)

**DOI:** 10.1186/s41687-019-0126-1

**Published:** 2019-06-07

**Authors:** Maiken Pontoppidan, Stefan Bastholm Andrade, Ingeborg Hedegaard Kristensen, Erik Lykke Mortensen

**Affiliations:** 1 VIVEs – The Danish Centre for Social Science Research, Herluf Trolles Gade 11, 1052 Copenhagen, Denmark; 2 Section for Nursing, Aarhus University, Aarhus, Denmark; 3 Section of Occupational and Environmental Health, University of Copenhagen, Copenhagen, Denmark

**Keywords:** Parenting, Confidence, Child, Infant, Family, Assessment, First-time mothers

## Abstract

**Background:**

Parenting confidence is a key factor in predicting a range of outcomes for both parents and children, such as parental depression, parental stress, and child health development. This study examines maternal confidence in at-risk and not-at-risk mothers and the psychometric properties of the Karitane Parenting Confidence Scale (KPCS).

**Results:**

The total sample consisted of 695 mothers (488 not-at-risk and 207 at-risk) from a community setting. Cronbach’s alpha ranged from 0.72 to 0.79, and item-rest correlations ranged from 0.17 to 0.57. Total score improved significantly from 41.75 at two months to 42.41 at six months for the not-at-risk group and increased significantly from 39.51 at two months to 41.12 at six months for the at-risk group. The differences between the two risk groups were significant at both times.

**Conclusion:**

The KPCS has acceptable internal consistency, but an overall ceiling effect, with many items characterized by low discrimination. Despite a significant difference in maternal confidence between at-risk and not-at-risk mothers at both two and six months, the total score did not predict risk status very well in this sample. A nine-item version may be equal to the original 15-item version.

## Background

Infants are dependent on their parents’ ability to actively support their development, especially in the first few months of their life. Parenting confidence, or how parents perceive themselves in the parent role, is a key factor in predicting a range of parental and child outcomes such as parental depression, parental stress, and child development ([6, 17]; Montigny and Lacharite 2005).

Interventions aimed at improving parenting competencies, and thereby parenting confidence, are widely used in developed countries. To determine whether parents are in the target group for intervention and whether the intervention will improve their competencies, both clinicians and researchers must assess parenting confidence [9].

The concept of *perceived parental efficacy* is based on Bandura’s work on parental self-efficacy [2], and is defined as “beliefs or judgments a parent holds of their capabilities to organize and execute a set of tasks related to parenting a child” [12]. Črnčec and colleagues found three different approaches to measuring parenting confidence [11]: (1) scales employing task-specific items tailored to specific child ages, (2) scales employing general items not linked to specific parenting tasks, and (3) scales employing a global approach that views parenting confidence as a part of a more stable personality trait that influences a range of tasks. As no task-specific assessment instrument was available in Danish for health professionals for measuring parenting confidence in parents of infants, we decided to translate and validate such an instrument for use in both research and clinical practice, namely the Karitane Parenting Confidence Scale (KPCS) [9]. The KPCS was chosen because it was developed for clinical use with new families and specifically targets areas that new mothers worry about such as eating, sleeping and crying.

### The Karitane parenting confidence scale (KPCS)

The KPCS was developed in 2008 by Rudi Črnčec, Bryanne Barnett, and Stephen Matthey for the Australian organization Karitane [9]. The scale, which is based on attachment theory, builds on a strengths-based relationship, in which the focus is on acknowledging the parents’ strengths and their intimate relationship with their child. Grounded in Bandura’s self-efficacy theory, the KPCS is a task-specific scale for measuring perceived parenting self-efficacy (PPSE) [3, 11]. The KPCS covers the following themes that emerged through focus groups with parents and professionals: feeding, settling, establishing sleep routines, interpreting cries and cues, playing, communicating, responding to needs, management of minor illness, providing a stimulating environment and support from the partner. Designed to be simple to administer, complete, and score, it is therefore easy to use for both researchers and practitioners working within a clinical setting with parents of infants up to 12 months old [9].

The KPCS consists of 15 items, scored on a four-point scale (*No, hardly ever; No, not very often; Yes, some of the time; Yes, most of the time*). Cronbach’s alpha is 0.81, and the test-retest reliability is 0.88, with a 28-day retest interval [10]. The possible score range is 0–45, with high scores being favorable. Suggested clinical cut-off scores are as follows: severe clinical range < 31, moderate clinical range 31–35, mild clinical range 36–39, and non-clinical range ≥ 40. An improvement of six points or more indicates a reliable change [9].

The validation sample consisted of 187 mothers aged 18 years or more with an infant aged below 12 months. The sample comprised a community control group (*n* = 47), an early intervention group (*n* = 42) with self-referred women who participated in a parenting class, a moderate difficulties group (*n* = 55) with mothers referred to an outpatient program, and a major difficulties group (*n* = 53) with mothers attending a residential parenting program [9]. The mean age of the mothers was 32.0 years, the mean infant age was 24.7 weeks, and mothers had a mean of 1.5 children. In all, 8% of the mothers had not completed a university or vocational education. Apart from the initial validation study, based on a relatively small sample of mothers (*N* = 187) by the developers [9, 10], no psychometric evaluation of the KPCS has been conducted.

The aim of this study is to evaluate the internal and external validity of the Danish version of the KPCS in a community sample of first-time mothers. We will also examine how scores change over time in both at-risk and not-at-risk groups. We hypothesize (1) that the KPCS will have acceptable internal consistency, test-retest reliability, and concurrent validity, (2) that at-risk mothers will have lower confidence than not-at-risk mothers, and (3) that maternal confidence will improve from child age 2 to 6 months.

## Methods

### Translation procedure

The KPCS was translated according to the World Health Organization’s (WHO) “Process of translation and adaptation of instruments.” The stages in this process consist of forward translation, expert panel, back-translation, pre-testing, cognitive interviewing, and a final version. The first and third authors independently forward translated the original scale and discussed the first draft with an expert panel consisting of two experienced researchers with in-depth knowledge of assessment instruments and infant development. As there is no direct Danish translation for “I am confident,” we mainly use one phrase in Danish but had to use other phrases on two occasions to make sure that the correct meaning of items was transferred to the Danish version. The first and third authors agreed on a second version, which was pre-tested with a small group of parents of infants. A third version with minor changes was pre-tested with cognitive interviews with a group of eight vulnerable parents. As the cognitive interviews indicated no need for further changes, the third version was back-translated by a native-speaking English researcher fluent in Danish. The back-translation necessitated small changes, leading to the final version of the instrument.

### Sample

The sample consists of 695 (67%) out of 1040 eligible first-time mothers residing in the Central Denmark Region [19]. Mothers participated in a quasi-experimental intervention study conducted in 2013–2014. All first-time mothers giving birth and who lived in a district with a health visitor participating in the study were included and invited to fill in questionnaire’s two and six months after birth when the health visitor paid her first ordinary home visit. Mothers were excluded if they had insufficient Danish language skills. Only mothers with no missing items on the KPCS at both the two- and six-month assessments were included in the present analyses. Of the full sample (*N* = 695) a total of 50 vulnerable mothers received an intervention during the study. Mothers who were eligible for intervention were defined as vulnerable based on having experienced either a moderate preterm birth (gestational age ≥ 32 < 37), moderate symptoms of depression (Edinburgh Postnatal Depression Scale (EPDS) ≥8 < 13), or low parenting confidence (KPCS < 40).

As the KPCS score was used to identify vulnerable mothers in the intervention study, we could not use the exact same inclusion criteria in this study. For the present study, the sample was divided into a not-at-risk and an at-risk sample. Mothers were included in the at-risk sample if they fulfilled at least one of the following criteria at baseline: (1) young mother, age < 20, (2) low education, grade 9 or 10, (3) symptoms of depression, EPDS ≥8, and (4) preterm birth, gestational age < 37 weeks. A total of 207 (30%) mothers were categorized as at-risk. Of the 50 intervention mothers, 26 were in the at-risk group and 24 in the not-at-risk group.

### Measures

To establish concurrent validity of the KPCS, mothers also completed the Parental Stress Scale (PSS) [5] and the Edinburgh Postnatal Depression Scale (EPDS) [7]. The PSS consists of 18 items rated on a five-point Likert scale. The total score ranges from 0 to 90 with a high score indicating higher levels of parental stress. Cronbach’s alpha is 0.83 [5]. The EPDS consists of 10 items rated on a four-point scale. The total score ranges from 0 to 30 with a high score indicating higher levels of maternal depression. Cronbach’s alpha is 0.87 [8].

### Statistical analyses

We calculated descriptive statistics (means and standard deviations) for all variables. We applied t-tests to test for differences for continuous variables (Mann-Whitney U test for independent groups and Wilcoxon signed-rank test for dependent groups) while applying Chi-square tests for categorical variables. We analyzed internal consistency with Cronbach’s alpha and test-retest reliability with Pearson correlation coefficients. Coefficients of 0.70 or higher are considered acceptable [15]. Concurrent validity was assessed with Pearson correlations between the KPCS score and the PSS and EPDS scores. We expected medium sized negative correlations between the KPCS and both the PSS and EPDS.

Initially, we conducted factor analyses and Rasch analyses to examine the structure of the data. As these analyses revealed a data structure characterized by a substantial ceiling effect and high local dependency between items, we did not conduct any further model based analyses.

To study changes in maternal confidence over time we examined in KPCS total scores in a linear regression model with indicator variables for the intervention group and risk status and test of interaction between risk status and intervention group. We also wanted to examine if the KPCS could discriminate between at-risk and not-at-risk groups and how well it predicted maternal risk-status. We applied linear discriminant analyses (LDA) to examine this.

To evaluate whether it was possible to create a more internally consistent and shorter scale, we examined the 15 items of the KPCS to determine whether any items provided only minimal information and thus could be left out. To do this we ranked all 15 items according to the mean, standard deviation, and ability to differentiate between either time or risk status. Drawing on these characteristics and the item-rest scores, we identified items that could be left out for a possible shorter version of the KPCS. The item-rest correlation is the correlation between an item and the scale that is formed by all other items (Nunnally and Bernstein 1994). We used STATA 14 and R 3.2.2 for data analyses.

## Results

### Characteristics

Table 1 presents the demographic characteristics of the sample 2 months after birth.

**Table 1:** Demographic characteristics of not-at-risk and at-risk mothers 2 months after birth

	All (*n* = 695)		Not-at-risk (*n* = 488)		At-risk (*n* = 207)	
	Mean	SD	Mean	SD	Mean	SD
Mother age***^a^	30.14	4.00	30.54	3.87	29.09	4.15
Gestational age in weeks	39.71	1.74	40.05	1.16	38.94	2.47
EPDS (0–30)	4.59	3.36	3.46	2.00	7.14	4.29
PSS (0–90)	32.38	7.78	31.20	6.70	35.16	9.33
Background variables	N	%	N	%	N	%
Smoker**^b^	28	4	12	2	16	8
Non-smoker**^b^	658	95	471	97	187	90
No information	9	1	5	1	4	2
Boy	340	49	228	47	112	54
Girl	350	50	255	52	95	46
No information	5	1	5	1	1	0
Short education (grade 9 or 10)	85	12	0	0	85	41
Long education (>grade 10)	608	87	486	100	122	59
No information	2	0	2	0	0	0

*** *p* < 0.000; ** *p* < 0.01; * *p* < 0.05; ^a^ = independent t-test comparing at-risk and not-at-risk mothers; ^b^ = Chi-square test comparing at-risk and not-at-risk mothers

The mothers in the at-risk sample were significantly younger and smoked significantly more than not-at-risk mothers. As low education was a criterion for becoming an at-risk mother, the at-risk mothers also had less education.

### Prevalence of clinical KPCS

Table 2 shows the prevalence of the clinical levels of mothers’ parenting confidence.

**Table 2:** Clinical levels of KPCS scores according to risk status and infant age at the time of assessment

	All				At-risk				Not-at-risk			
	2 months		6 months		2 months		6 months		2 months		6 months	
	N	%	N	%	N	%	N	%	N	%	N	%
Non-clinical range	511	74	596	86	113	55	161	78	398	82	435	89
Mild clinical range	139	20	78	11	62	30	32	15	77	16	46	9
Moderate clinical range	35	5	16	2	28	14	11	5	7	1	5	1
Severe clinical range	10	1	5	1	4	2	3	1	6	1	2	0
Total	695	100	695	100	207	100	207	100	488	100	488	100

Severe clinical range < 31, moderate clinical range 31–35, mild clinical range 36–39, and non-clinical range ≥ 40

Most mothers (74–86%) show levels of parent confidence in the non-clinical range. Mild clinical levels are found in 11–20%, whereas moderate to severe levels are found in 3–6% of mothers, depending on infant age at KPCS administration. Significantly more mothers in the at-risk group show clinical levels both at 2 and 6 months after birth compared to not-at-risk mothers (2 months: χ^2^ (1, *N* = 695) = 52.93, *p* < .000; 6 months: χ^2^ (1, N = 695) = 14.44, p < .000).

#### Internal validity

Table 3 shows the distribution of responses for the sample when the infant was two and six months old.

**Table 3:** Response distribution at two and six months n = 695

Response	2 months all					6 months all				
	0	1	2	3	Mean	0	1	2	3	Mean
1. Feeding baby	2	1	13	679	2.97	0	3	20	672	2.96
2. Settling baby	0	1	44	650	2.93	0	0	17	678	2.98
3. Establishing good sleep routine	1	44	244	406	2.52	2	34	214	445	2.59
4. Knowing what to do when baby cries	0	5	125	565	2.81	2	0	57	636	2.91
5. Understanding baby’s signals	0	6	242	447	2.63	1	7	170	517	2.73
6. Soothing baby when distressed	1	1	80	613	2.88	0	0	27	668	2.96
7. Playing with baby	0	10	138	547	2.77	0	7	104	584	2.83
8. Handling cold or minor illness	11	64	307	313	2.33	1	12	190	492	2.69
9. Confidence in support from partner	1	7	76	611	2.87	1	10	89	595	2.84
10. Baby is doing well	0	8	70	617	2.88	1	0	34	660	2.95
11. Making decisions about care of baby	0	2	44	649	2.93	0	0	18	677	2.97
12. Being a mother/father is very stressful	14	247	223	211	1.91	13	218	265	199	1.94
13. Feel doing a good job as mother/father	1	7	119	568	2.80	0	2	91	602	2.86
14. Other people believe doing a good job	0	0	29	666	2.96	0	0	29	666	2.96
15. Feel sure about support from others	0	3	65	627	2.90	0	4	85	606	2.87

Responses: 0: No, hardly ever; 1: No, not very often; 2: Yes, some of the time; 3: Yes, most of *the time*

Table 4 shows internal consistency for the KPCS at two and six months for the 15- and nine-item version. Cronbach’s alpha was acceptable, with only slight differences between assessments, ranging from 0.72 to 0.79. We define the correlation between each item and the sum of the remaining items as the item-rest correlation. Item-rest correlations ranged from 0.17 to 0.57. Although items 1, 9, 11, 14, and 15 had low item-rest correlations, these correlations were acceptable for the other ten items.

**Table 4:** Item-rest correlations and total Cronbach’s alpha for 15- and nine-item versions at two and six months

Item	2 months		6 months	
	Item-rest correlation		Item-rest correlation	
	15 items	9 items	15 items	9 items
1. Feeding baby	0.21	–	0.17	–
2. Settling baby	0.39	–	0.39	–
3. Establishing good sleep routine	0.49	0.49	0.41	0.39
4. Knowing what to do when baby cries	0.57	0.56	0.38	0.38
5. Understanding baby’s signals	0.51	0.53	0.47	0.46
6. Soothing baby when distressed	0.48	0.46	0.37	0.32
7. Playing with baby	0.37	0.35	0.38	0.38
8. Handling cold or minor illness	0.37	0.36	0.34	0.35
9. Confidence in support from partner	0.18	–	0.19	–
10. Baby is doing well	0.49	0.47	0.34	0.32
11. Making decisions about care of baby	0.36	–	0.24	–
12. Being a mother/father is very stressful	0.47	0.46	0.37	0.37
13. Feel doing a good job as mother/father	0.57	0.56	0.49	0.47
14. Other people believe doing a good job	0.29	–	0.31	–
15. Feel sure about support from others	0.23	–	0.33	–
Alpha	0.79	0.79	0.76	0.72

### The 9 item KPCS – internal validity

As several items show a mean close to the maximum value we wanted to examine if any of the existing 15 items could be left out to create a shorter and more internally consistent scale. Based on the item mean, standard deviation, item-rest score and ability to differentiate between either time or risk status we identified six items that provided only minimal information (items 1, 2, 9, 11, 14, and 15). We thus evaluated a nine-item version of the KPCS consisting of the following items: 3, 4, 5, 6, 7, 8, 10, 12, and 13. Table 4 presents the item-rest correlations and Cronbach’s alpha for both the nine- and 15-item versions. Although Cronbach’s alpha tends to increase with more items, the alphas for the nine-item version were identical to the 15-item version at two months, and only slightly lower at six months.

#### External validity

Table 5 shows the means, standard deviations, and differences between and within groups for nine- and 15-item versions at two and six months. The mean KPCS score for the entire sample was 41.08 (SD 3.37, skewness − 1.34, kurtosis 2.37, range 25–45) at two months and 42.03 (SD 2.69, skewness − 1.52, kurtosis 3.42, range 27–45) at six months. The correlation between the KPCS total scores at two and six months was 0.62 for the whole sample. The score at six months was significantly higher than the score at two months. For the nine-item version, mothers improved significantly from 23.52 at two months to 24.45 at six months.

**Table 5:** KPCS means, standard deviations, and differences between and within groups for nine- and 15-item versions at two and six months

	2 months		6 months		Δ	
	Mean	SD	Mean	SD	Mean	SD
15-item version (0–45)						
All	41.08	3.37	42.03	2.69	0.95***	2.72
Not-at-risk	41.75	2.86	42.41	2.37	0.67***	2.26
At-risk	39.51	3.92	41.12	3.15	1.60***	3.49
Group Δ	1.7***		0.99***		−0.93**	
9-item version (0–27)						
All	23.52	2.87	24.45	2.23	0.93***	2.37
Not-at-risk	24.03	2.49	24.74	1.97	0.72***	2.00
At-risk group	22.33	3.22	23.75	2.63	1.42***	3.02
Group Δ	1.70***		0.99***		−0.70**	

*** *p* < 0.000; ** *p* < 0.01; * *p* < 0.05

Δ: Difference between means at two and six months. Positive value indicates higher score at six months

Group Δ: Difference between at-risk and not at risk group. A positive value indicates a higher score in the not-at-risk group

For both groups, there was a ceiling effect on the total KPCS score, especially at six months. Although skewness and kurtosis were moderate for the at-risk group (skewness − 0.67, kurtosis 0.10 at two months and − 1.28 and 2.09, respectively, at six months), they were more problematic for the not-at-risk group (skewness − 1.73, kurtosis 5.29 at two months and − 1.51 and 3.65 respectively at six months). Many items had mean values well above 2.80 and standard deviations around 0.3, for both the not-at-risk and at-risk samples. The mean of this sample is consistent with the mean of the control group (approximately 42) as reported in the original study [10], indicating that a ceiling effect was also present in that study.

### The 9 item KPCS – external validity

The range for the nine-item version is 0–27, and the correlations between the nine- and 15-item versions are 0.98 at two months and 0.96 at six months (Table 5). The mean for the not-at-risk group significantly increased from 24.03 at two months to 24.74 at six months, and from 22.33 at two months to 23.75 at six months for the at-risk group. The difference between the not-at-risk and at-risk group was significant at both two and six months and improvement over time was significantly larger for the at-risk group than for the not-at-risk group. Skewness and kurtosis were marginally better for the nine-item version than for the 15-item version.

### Concurrent validity

We established concurrent validity by examining the correlations between the KPCS total score and the total scores of parental stress (PSS) and depression (EPDS) at both times. The correlation between KPCS and PSS was − 0.65 at two months and − 0.64 at six months. For KPCS and EPDS, the correlations were − 0.60 at two months and − 0.61 at six months.

### Change over time

While the mean of not-at-risk mothers was 41.75 at two months, it increased significantly to 42.41 at six months (Table 5). The increase was significant when we controlled for intervention status (*p* < 0.000), and there was no interaction between risk status and intervention status (*p* = 0.23). The mean of the at-risk mothers was 39.51 at two months, increasing significantly to 41.12 at six months. At two months, the mean of the at-risk group was equal to the mean of the moderate difficulties group in the original study but higher than that of the clinical groups [10]. While the at-risk group mean is in the mild clinical range at two months, it is in the non-clinical range at six months.

### Discriminative validity - differences between at-risk and not-at-risk groups

When comparing the means of the at-risk and the not-at-risk groups at both time points, we find a significant difference between total score at both two months (2.24-point difference) and six months (1.29-point difference). Although the not-at-risk group was significantly more confident than the at-risk group at six months, the at-risk mothers improved significantly more over time. Intervention mothers improved significantly more than those who did not receive any intervention.

**Fig. 1:**
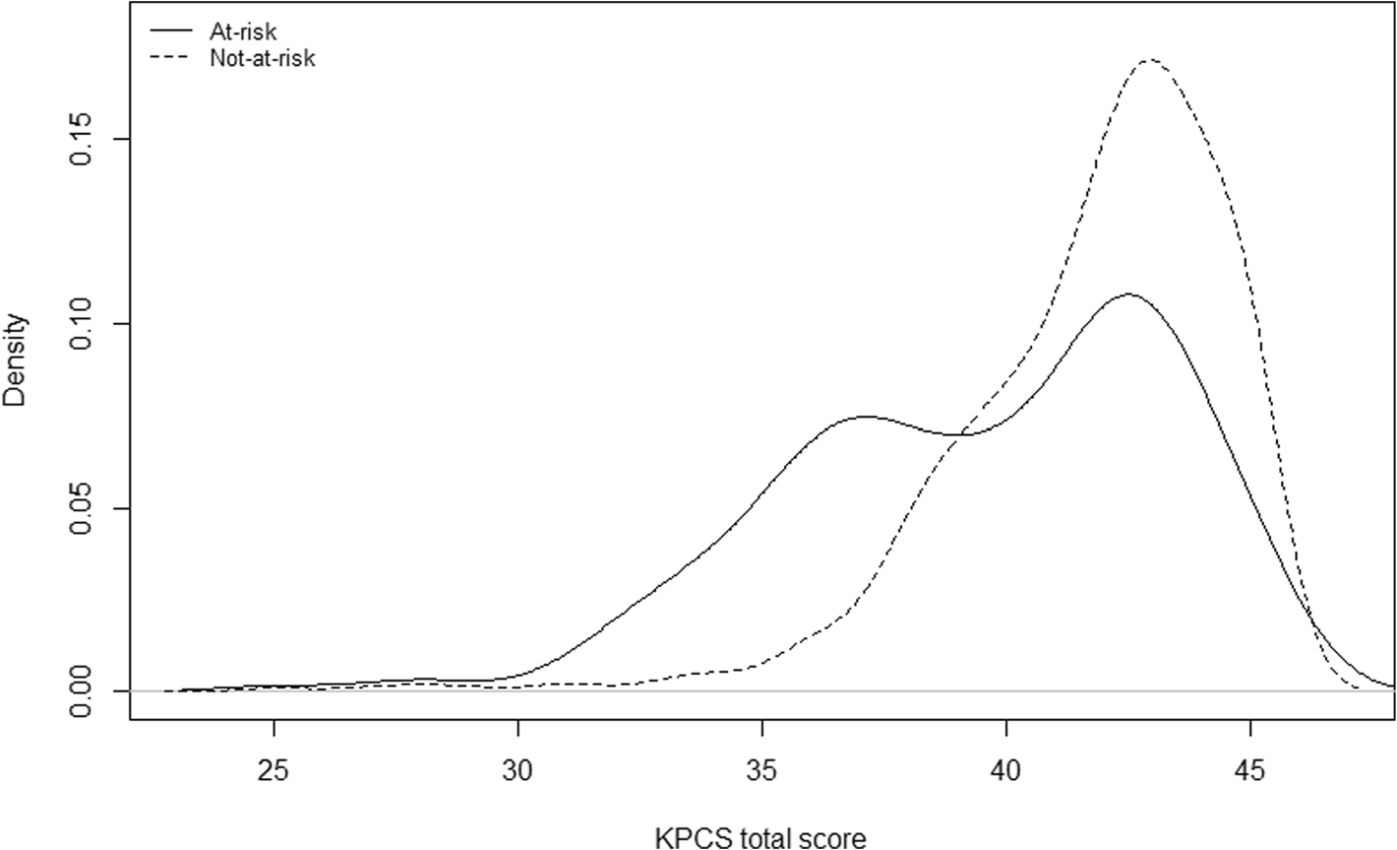
Density plot of KPCS mean scores for at-risk and not-at-risk mothers at two months

**Fig. 2:**
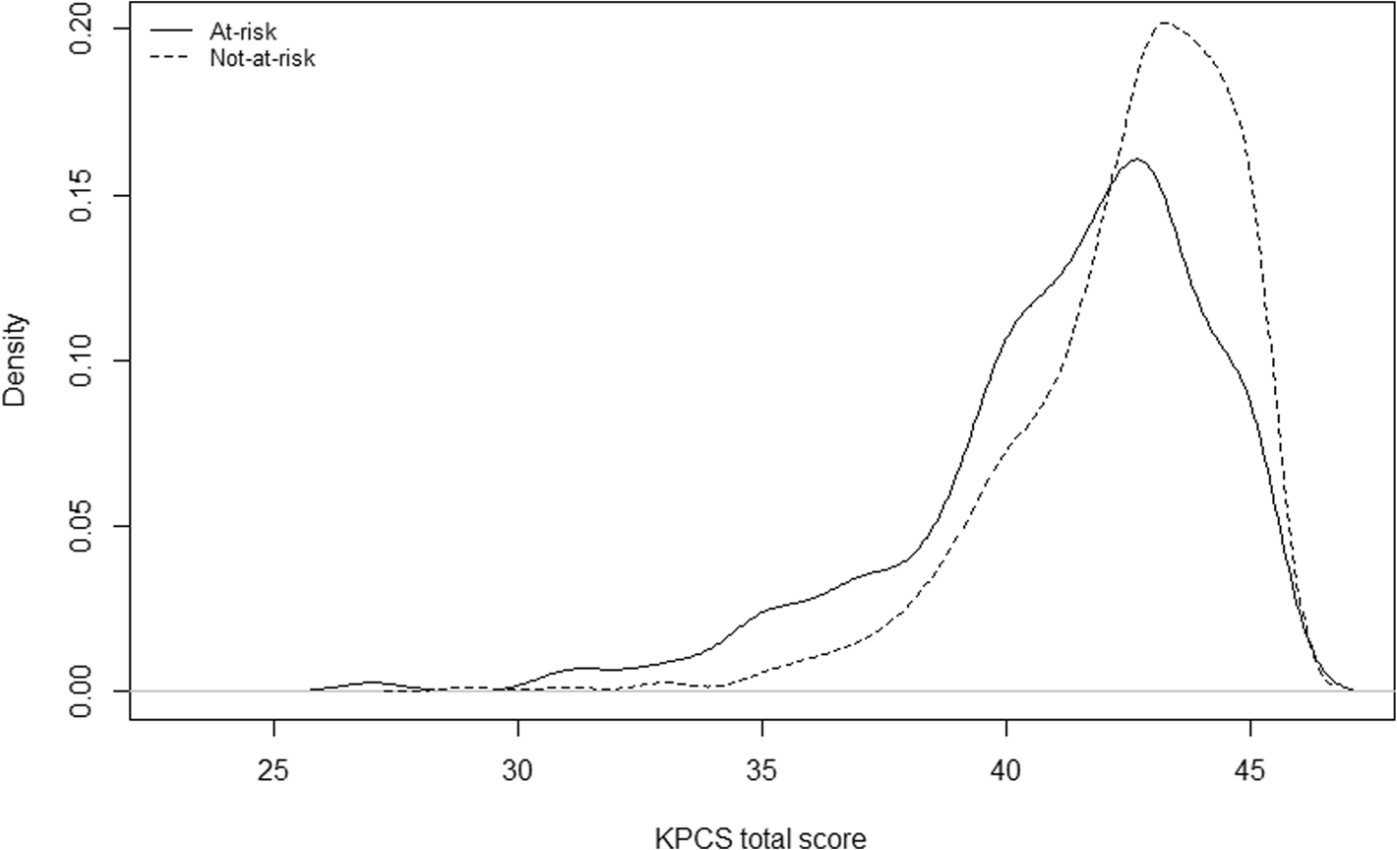
Density plot of KPCS mean scores for at-risk and not-at-risk mothers at six months

Figures 1 and 2 show the density plots of the not-at-risk and at-risk groups at two and six months. The distribution of mean KPCS total score at two and six months for the at-risk at not-at-risk groups show a large overlap, especially at six months.

Table 6 shows the predicted versus the observed risk status at two months for both 15- nine-item versions based on linear discriminant analysis. While the agreement is high for the not-at-risk mothers, it is low for at-risk mothers, where 22–24% are correctly predicted as belonging to the at-risk group.

**Table 6:** Predicted and observed risk status at two months for 15- and nine-item versions based on linear discriminant analysis

Predicted	2 Months 15 Items		2 Months 9 Items	
	Observed		Observed	
	Not-at-risk	At-risk	Not-at-risk	At-risk
Not-at-risk	466 (95%)	158 (76%)	468 (96%)	162 (78%)
At-risk	22 (5%)	49 (24%)	20 (4%)	45 (22%)
Total	488	207	488	207

## Discussion

In this study, we examined the internal and external validity of the KPCS and studied changes over time in first-time mother’s maternal confidence. Internal consistency was acceptable, but many items showed small variance, and both the item scores and the total score showed ceiling effects. Concurrent validity was expressed as a medium to a high negative relationship, with both parental stress (PSS) and depression (EPDS) in concordance with the previous study [10] and our hypothesis. The nine-item version seems to work as well as the original 15 item version. The shortened version can, therefore, be used to place less burden on the mothers. Most mothers showed non-clinical levels of maternal confidence at both time points; only 1% showed severe clinical levels. As expected, significantly more families in the at-risk group compared to the not-at-risk group show clinical levels at both two and six months. A small number of families in the not-at-risk group do, however, show some level of clinical problems at both assessments. As the clinical levels based on Australian data may not be applicable to a Danish population, they must be interpreted with caution. The two cultures may differ regarding family norms, support offered to families with infants, parental leave, and other issues that may well influence parental confidence.

### Ceiling effect

Both the nine-item and 15-item version of the KPCS showed high mean scores and relatively high skewness and kurtosis. Skewed distributions are not unusual for this type of measure [11], as they are also found with, for example, measures of depression [13] and other mental symptoms [4]. As these measures are constructed for capturing a concept that is present in only a few parents, they are almost inherently skewed when applied to a community sample. Therefore, whether the observed skewness reflects the actual distribution of parenting confidence or rather item content and selection remains an open question.

The KPCS’s four response options (*No, hardly ever; No, not very often; Yes, some of the time; Yes, most of the time*) may also contribute to the ceiling effect. During the translation phase, some parents pointed out that they would have preferred being able to choose the options *Yes, always* or *No, never*. The omission of these options likely reduced the variation in responses. As parents said that the existing response options were not adequate, either revising the response options or adding more may be important.

The items in the KPCS may not fully capture allessential aspects of parent worries in relation to infant care, perhaps partly explaining the ceiling effect. If so, then constructing new items or rewording existing ones could improve the scale properties and make the ceiling effect less pronounced. For example, issues in relation to feeding—e.g. how to recognize hunger cues, how often and how much the infant should eat—are sources of worry for mothers of infants [20, 23]. However, item 1 (“I am confident about feeding my baby”) does not seem to capture the variance in feeding-related worries, as the mean score is 2.95–2.98. Thus item 1 should either be rephrased or supplemented by new items related to infant feeding.

Despite the observed skewness of the KPCS scores, distinguishing between a not-at-risk and at-risk group in the study sample remained possible. For intervention studies, a ceiling effect is problematic, as a very high score at baseline makes improving over time difficult. However, the KPCS has been used as an outcome in several other studies of parenting interventions for infants, with significant improvement in total scores over time [14, 16, 24].

### Changes over time

Mothers generally felt less confident at two months than at six months. This finding is consistent with other research showing that the earliest months as a parent can be stressful and challenging, especially concerning difficulties in soothing and comforting the infant [20], infant crying [25], and sleep disturbances [1, 21].

Standard care in Demark represents a relatively comprehensive intervention as families are entitled to five to six free visits from a health visitor and three free child-health visits to a general practitioner within the first year after birth [26]. These visits are universally offered to all families—both at-risk and not-at-risk. More visits can be offered if the health visitor deems them necessary. The improvement in KPCS score over time for both the at-risk and the not-at-risk groups may be due to the relatively intensive usual care that all Danish mothers receive. Whether the KPCS score improves over time in countries where the usual care is less intensive is for future research to determine.

### Discriminative validity

At-risk mothers felt significantly less confident than not-at-risk mothers at both assessments which is consistent with previous research that found that mothers with more risk factors generally had lower self-efficacy than other mothers [11, 18]. Nonetheless, we find that at-risk mothers improved significantly more over time than not-at-risk mothers and that the difference between the two groups was reduced by more than 50% over time. Thus the at-risk group is catching up with the not-at-risk mothers, even though the difference between the two groups remains significant at six months.

The large overlap in the KPCS total score distributions for the at-risk and not-at-risk groups at both two and six months indicates that the total score does not predict at-risk status well for this sample and that the KPCS may not be able to discriminate between at-risk and not-at-risk status. This could be caused by ceiling effects specific to this sample, but could also be because the risk factors used to generate the at-risk group do not capture all aspects of being a vulnerable mother. Interestingly, of the 50 intervention mothers, only 26 were in the at-risk group. While the 24 intervention mothers in the not-at-risk group all had clinical levels of KPCS scores, they had none of the risk factors used for defining the at-risk group. This finding shows that if only risk factors are used for identifying mothers for intervention, a considerable group of mothers with low parenting confidence (according to the KPCS) will not be selected for intervention.

### Limitations

The current study has some limitations. First, we did not include a clinical group of mothers and therefore do not know how mothers with lower levels of parent confidence would score or change over time. Second, as the intervention study was aimed at mothers, and no data on fathers was collected, the sample included only mothers. Moreover, as the original KPCS study included only mothers, how this instrument works with fathers remains unknown. Although a few fathers were included in our pre-test with cognitive interviewing in the translating phase, aside from their comments on the first suggestion for the Danish title of the measure, they did not express any concerns about the KPCS. Third, the intervention study included only first-time mothers. However, as mothers with more than one child have more parental experience, we would expect them to have higher levels of parent confidence than first-time mothers [22].

## Conclusion

In sum, while the internal consistency of the KPCS is acceptable, an overall ceiling effect exists, and many items are characterized by low discrimination. All mothers improved their confidence over time, and at-risk mothers improved significantly more than not-at-risk mothers. Despite a significant difference between at-risk and not-at-risk mothers at both two and six months, the total score did not predict risk status very well. We evaluated a reduced nine-item version of the KPCS and found it to be as good as the original 15-item version.

## Data Availability

The datasets generated and analyzed during the current study are not publicly available due to confidentiality issues but are available from the corresponding author on reasonable request.

## Abbreviations

EPDS: Edinburgh Postnatal Depression Scale
KPCS: Karitane Parenting Confidence Scale
LDA: Linear discriminant analyses
PPSE: Perceived Parenting Self-efficacy
PSS: Parental Stress Scale
WHO: World Health Organization
